# Impact of dipeptidyl peptidase I and neutrophil serine proteases on neutrophil functional responses

**DOI:** 10.3389/fphar.2026.1689804

**Published:** 2026-04-24

**Authors:** Dedong Li, Kuan-Ju Chen, Nandita Niranjan, Jessica Basso, Daniel LaSala, Mei-Fong Pang, Craig Smith, Tam Nguyen, Vanessa de Carvalho Oliveira, David C. Cipolla, Patrick P. McDonald

**Affiliations:** 1 Department of Research, Insmed Incorporated, Bridgewater, NJ, United States; 2 Immunology Graduate Program, Faculty of Medicine, Université de Sherbrooke, Sherbrooke, QC, Canada

**Keywords:** bactericidal activity, brensocatib, cathepsin C, NETosis, oxidative burst, phagocytosis

## Abstract

Dipeptidyl peptidase-1 (DPP1) is a lysosomal cysteine protease essential for activating neutrophil serine proteases (NSPs), including neutrophil elastase, cathepsin G, and proteinase 3, during neutrophil differentiation in the bone marrow. Because NSP-mediated tissue damage contributes to chronic inflammatory and autoimmune diseases, targeting NSPs has emerged as a therapeutic strategy. In this regard, small molecule drugs have been developed such as brensocatib–a competitive, reversible DPP1 inhibitor. Active DPP1 is expressed in mature neutrophils, which raises the possibility that DPP1 inhibition might affect more than just NSP activities. Here, we investigated the effects of DPP1 and NSP inhibition or ablation on neutrophil function using brensocatib, as well as *DPP1* ^*−/−*^ and triple NSP knockout (*NE* ^*−/−*^ *CatG* ^*−/−*^ *Pr3* ^*−/−*^) mice. As expected, DPP1 inhibition with brensocatib during mouse bone marrow hematopoietic stem cell differentiation dose-dependently reduced NSP activities. While DPP1 inhibition or ablation suppressed neutrophil extracellular trap (NET) formation in mice, it had no significant impact on granulocytic differentiation, migration, phagocytosis, reactive oxygen species production, or bacterial killing. A similar outcome was observed in triple NSP KO mice. In mature human neutrophils, brensocatib had no effect on any of these responses, including NET formation. These findings suggest that while DPP1 is crucial for NSP activation during early neutrophilic differentiation, it does not substantially influence differentiation itself or core neutrophil functions, except for NET formation in mice. This study advances our understanding of the roles of DPP1 and NSPs in neutrophil biology and further emphasizes the high selectivity of brensocatib in the main target cells.

## Introduction

Neutrophils are pivotal components of the innate immune system, serving as first responders to microbial pathogens and inflammation. Upon infection or tissue injury, neutrophils are rapidly recruited from the bloodstream and mount a number of responses to eliminate non-self-particles (e.g., microbes, cell debris, inorganic matter); these responses notably include phagocytosis, reactive oxygen species (ROS) production, and the formation of neutrophil extracellular traps (NETs) (Zhang et al., 2024; Herro and Grimes, 2024; Burn et al., 2021). Likewise, activated neutrophils can exocytose a host of antibacterial products and lytic enzymes from their intracellular granules. As part of this process, termed degranulation, the cells release neutrophil serine proteases (NSPs) from their azurophilic granules, which are the least easily mobilized; NSPs mainly include neutrophil elastase (NE), cathepsin G (CatG), and proteinase 3 (PR3) (Pham, 2006; Pham, 2008). These powerful proteolytic enzymes degrade microbial proteins and are essential for effective pathogen clearance and regulation of inflammation (Pham, 2006; Pham, 2008). Conversely, when in excess, the action of NSPs also contributes to the tissue damage that accompanies inflammatory reactions, both acute and chronic. As a result, inhibiting NSP activities to bring them back into balance with their cognate antiproteases represents an increasingly advocated therapeutic strategy (Chitsamankhun et al., 2024; Chalmers et al., 2023; Shen et al., 2021; Chalmers et al., 2025a).

Dipeptidyl peptidase I (DPP1), also known as cathepsin C, is a lysosomal cysteine protease responsible for excising dipeptides from the N-terminus of protein substrates (Pham, 2006; Pham, 2008; Pham and Ley, 1999; Adkison et al., 2002). DPP1 cleaves a propeptide sequence from NSP zymogens, converting them into their active forms during the promyelocyte stage of neutrophil maturation in the bone marrow. Genetic ablation or pharmacological inhibition of DPP1 results in the loss of NSP activity, as unprocessed NSP zymogens are degraded (Adkison et al., 2002; Basso et al., 2023). Brensocatib is an oral DPP1 inhibitor that demonstrates competitive, reversible and selective DPP1 inhibition (Doyle et al., 2016). By targeting DPP1, brensocatib exerts potent inhibitory effects on the activity of all three major NSPs, in both humans and rodents (Basso et al., 2023; Cipolla et al., 2023; Chalmers et al., 2020). Accordingly, brensocatib has displayed significant therapeutic effects in neutrophil-driven inflammatory disease rodent models, such as rheumatoid arthritis (McDonald et al., 2023). Likewise, the landmark Phase 3 ASPEN trial illustrated brensocatib’s efficacy and safety in patients over 12 with non-cystic fibrosis bronchiectasis (hereafter referred to as NCFB) (ClinicalTrials.gov number: NCT04594369) (Chalmers et al., 2025b). Notably, the earlier Phase 2 trial (WILLOW) had highlighted brensocatib’s immunomodulatory effects, which include reduced NSP activity, increased antimicrobial peptides, and alterations in inflammatory cytokines, underscoring an action that extends beyond protease inhibition (Johnson et al., 2025). Furthermore, brensocatib has shown therapeutic potential in other disease models, including lupus nephritis (McDonald et al., 2023; Chen et al., 2023), breast cancer lung metastasis (Xiao et al., 2021), rheumatoid arthritis (McDonald et al., 2023), and drug-induced liver injury (Raith et al., 2024). In this context, the actions of brensocatib have naturally prompted investigations into whether it might affect other processes beyond NSPs. For instance, granzyme B is a DPP1 substrate (Pham and Ley, 1999) that could, in principle, be affected by brensocatib; however, a recent study found that brensocatib does not deleteriously impact the *in vitro* killing capacity of human cytotoxic T lymphocytes (CTL) and natural killer (NK) cells (Sutton et al., 2024). Similarly, neutrophils are the main cellular target of brensocatib, but it remains unclear whether it affects neutrophil responses beyond abrogating NSPs, for instance, migration, phagocytosis, ROS production, and NET formation. Such a possibility stems from the observation that mature neutrophils can release active DPP1 (Chalmers et al., 2023; Hamon et al., 2016), which could in principle feed back on the cells to influence the above responses.

In this study, we investigated the effects of DPP1 inhibition on neutrophil biology, focusing on its role during maturation in hematopoietic stem cells (HSCs) from mice and humans, as well as its impact on mature neutrophils. To achieve this, we employed both genetic and pharmacological approaches in mouse bone marrow (BM) HSCs: DPP1 knockout (DPP1-KO) mice were used to assess the effects of genetic ablation, while brensocatib was used to evaluate pharmacological inhibition of DPP1. Additionally, BM HSCs from triple NSP knockout (triple NSP-KO, *NE* ^*−/−*^ *CatG* ^*−/−*^ *Pr3* ^*−/−*^) mice served as a comparative tool to examine the downstream effects of NSP deletion. To explore DPP1 inhibition in human cells, a CD34^+^ HSC differentiation system was utilized. Furthermore, the direct impact of DPP1 inhibition in mature neutrophils was assessed using primary human polymorphonuclear neutrophils (hPMNs). In all of the above systems, we systematically assessed neutrophil differentiation, migration, phagocytosis, ROS production, and NET formation. Our findings extend our understanding of the high selectivity of brensocatib toward neutrophil responses in inflammatory diseases.

## Results

### Granulocytic differentiation is not impaired by DPP1 inhibition or NSP deletion

**FIGURE 1:**
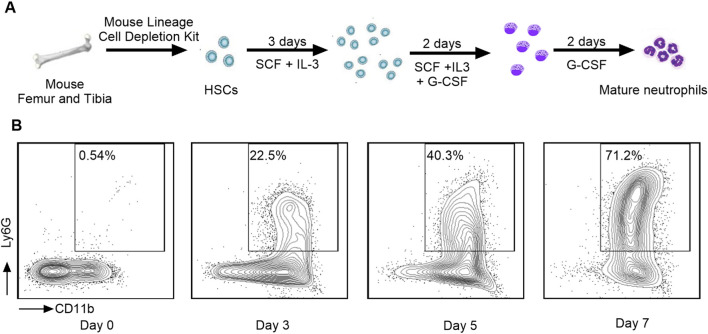
Mouse bone marrow progenitor cell-derived neutrophil differentiation protocol and surface marker analysis. **(A)** Schematic representation of the mouse neutrophil differentiation protocol. **(B)** At the indicated times (d0, d3, d5 or d7), cells were collected and stained with Live/Dead dye, anti-CD45, anti-CD11b, and anti-Ly6G. Flow cytometry was used to gate neutrophils (CD45^+^CD11b^+^Ly6G^+^).

We first differentiated mouse BM HSCs into mature neutrophils over a 7-day period (Figure 1A) in the presence of hematopoietic growth factors including stem cell factor (SCF), interleukin-3 (IL-3), and granulocyte colony-stimulating factor (G-CSF) (Gupta et al., 2014), which are essential for promoting myeloid differentiation and neutrophil lineage commitment. In this system, a progressive increase in the expression of neutrophil surface markers, including CD45, CD11b, and Ly6G, was observed. By day 7, flow cytometry analysis revealed that over 70% of the cells exhibited mature neutrophil markers, confirming differentiation efficiency (Figure 1B).

**FIGURE 2:**
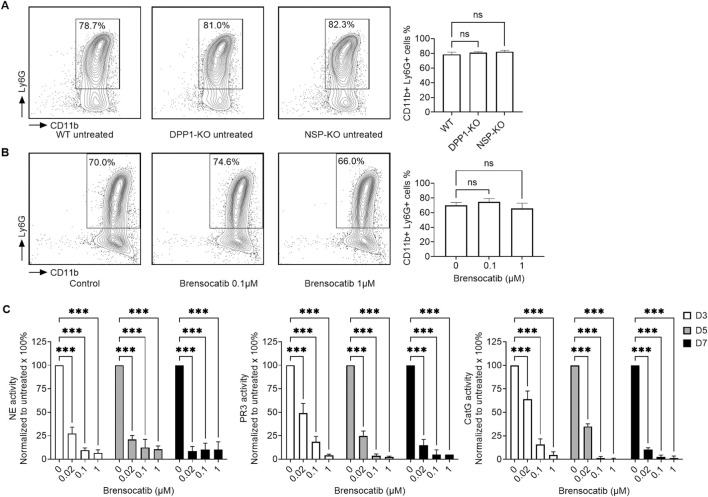
Effect of genetic ablation of DPP1 or all three major NSPs, or of brensocatib treatment, on mouse BM progenitor cell-derived neutrophil differentiation and NSP enzymatic activities. **(A)** Bones from WT, DPP1 knockout (KO), and triple NSP-KO mice were extracted. BM progenitor cells were isolated and differentiated into neutrophils. Flow cytometry analyses of neutrophils were conducted after 7 days of differentiation. **(B)** Brensocatib was added to the cells on days 0, 3, and 5 during neutrophil differentiation *ex vivo*, and neutrophils were analyzed by flow cytometry on day 7. **(C)** BM progenitor cell-derived differentiated neutrophils on days 3, 5 and 7 were collected, and NSP enzymatic activities in the cell lysates were measured. All graphed data is mean ± SEM from 3 independent experiments. Statistical analysis by one-way ANOVA **(B)**, and two-way ANOVA **(C)**. ***p < 0.001.

We subsequently investigated the impact of DPP1 inhibition, achieved through genetic ablation or pharmacological intervention, as well as through NSP deletion, on both neutrophil differentiation efficiency and NSP activities throughout the differentiation process. As shown in Figures 2A,B, neither genetic ablation of DPP1 nor of all three major NSPs (NE, PR3, and CatG) significantly affected neutrophil differentiation efficiency of mouse HSCs. Similarly, treatment with brensocatib at 0.1 µM or 1 µM throughout the 7-day differentiation period did not lead to notable changes in differentiation efficiency. By day 7, comparable differentiation rates (>70%) were observed across all groups. However, when NSP activities were assessed in these cells, a dose- and duration-dependent reduction in NSP activities was observed with brensocatib treatment. At both 0.1 µM and 1 μM, brensocatib led to a near-complete inhibition of all three NSP activities, with over 85% inhibition compared to the vehicle control, regardless of treatment duration (Figure 2C).

To further explore the effect of DPP1 inhibition on granulocytic differentiation, human CD34^+^ HSCs were differentiated into neutrophils using a 16-day protocol (Supplementary Figure S1A). Consistent with observations made in mouse neutrophils, treatment with either 0.1 µM or 1 µM of brensocatib for 9 days did not significantly affect the differentiation efficiency of human CD34^+^ HSC-derived neutrophils (Supplementary Figure S1B), while NSP activities were markedly suppressed (Supplementary Figure S1C).

### Neutrophil migration is not affected by DPP1 inhibition or NSP deletion

**FIGURE 3:**
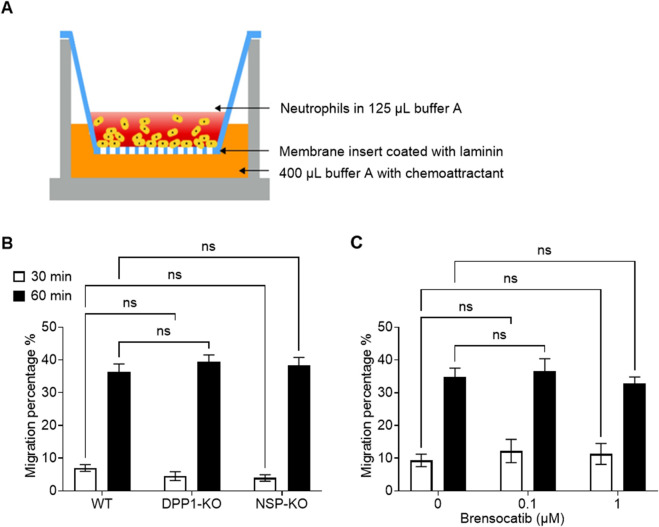
Genetic ablation of DPP1 or of all three major NSPs, or DPP1 inhibition, does not affect mouse neutrophil migration ex vivo. **(A)** Schematic representation of the transwell migration assay. **(B)** BM progenitor cell-derived neutrophils were loaded in the upper chamber of 3-µm pore size transwell plates, with 100 ng/mL CXCL2 in the lower chamber and 2 µg/cm2 laminin on the insert. Cells were further incubated at 37 °C for 30 or 60 min, and neutrophil migration rates were calculated as the # cells in the lower chamber/load control x100%. **(C)** Mouse BM progenitor cells from WT animals were treated with brensocatib on days 0, 3, and five at the indicated concentrations, or with vehicle control, while differentiating into neutrophils. On day 7, neutrophil migration was assessed as described in panel **(B)**. Data is mean ± SEM from 4 independent experiments. Statistical analysis by two-way ANOVA **(B,C)**.

Next, we investigated the impact of DPP1 inhibition and NSP deletion on neutrophil migration using a transwell culture system (Figure 3A). As shown in Figure 3B, after 30 or 60 min of incubation, neutrophils from DPP1-KO and triple NSP-KO mice exhibited migration rates similar to those of wild-type (WT) controls. Similarly, pharmacological inhibition of DPP1 with brensocatib during granulocytic differentiation did not affect the migration of the resulting neutrophils towards chemoattractants (Figure 3C).

**FIGURE 4:**
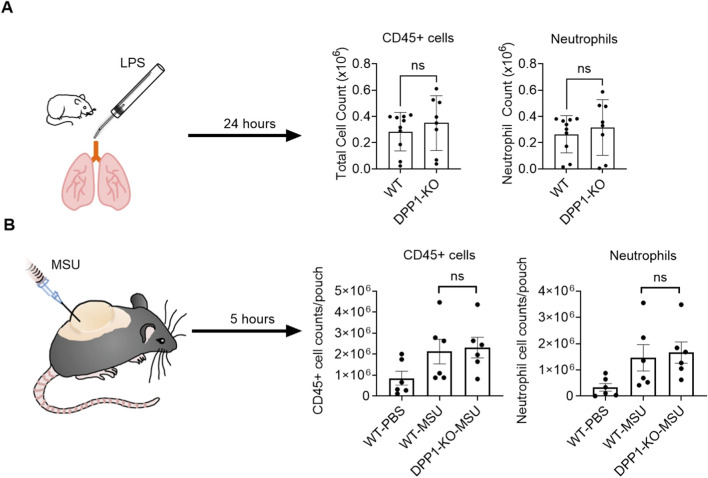
Genetic ablation of DPP1 has no impact on neutrophil migration or pro-inflammatory cytokine generation in vivo. **(A)** WT or DPP1-KO animals were instilled intratracheally with LPS or PBS control; after 24 h, BALF was collected, and infiltrating cells were analyzed by flow cytometry. **(B)** Sterile air was injected dorsally into WT or DPP1-KO animals to form a dorsal air pouch. Thereafter, MSU or PBS control was injected into the air pouch; after 5 h, the pouch was washed with PBS and infiltrating cells were analyzed by flow cytometry. Data is mean ± SEM from 6 to 10 animals in each group. Statistical analysis by Student’s t-test **(A)** and two-way ANOVA **(B)**.

To further examine the effect of DPP1 inhibition on neutrophil chemotaxis *in vivo*, we employed two inflammation models in mice: a lipopolysaccharide (LPS) challenge induced acute lung injury model and a monosodium urate (MSU) crystal-driven dorsal air pouch model. As shown in Figures 4A,B, genetic ablation of DPP1 did not affect the recruitment of CD45^+^ hematopoietic cells (including neutrophils) to the inflammatory site. Further analysis revealed that DPP1 deletion also had no significant effect on the levels of key pro-inflammatory cytokines (including IL-6, IL-1β, TNFα), or on lactate dehydrogenase (LDH) activity–a tissue damage marker (Supplementary Figure S2).

We similarly evaluated the effect of DPP1 inhibition on the granulocytic differentiation of human CD34^+^ progenitors, focusing on the ability of the resulting neutrophils to migrate towards a chemoattractant. As shown in Supplementary Figure S3A, brensocatib had no impact on migration. Stimulated neutrophils can release active DPP1 (Chalmers et al., 2023; Hamon et al., 2016) and accordingly, we detected DPP1 activity in mature neutrophils (Supplementary Figure S3B). We therefore investigated whether mature neutrophil migration might be affected by DPP1 inhibition. As shown in Supplementary Figure S3C, pretreating peripheral blood human neutrophils with brensocatib for 30 min did not alter their ability to migrate in a transwell assay. Supplementary Figure S3C also illustrates the superior ability of mature neutrophils to migrate, relative to the CD34^+^ HSCs that were differentiated into neutrophils (Supplementary Figure S3A).

### Neutrophil phagocytosis or bacterial killing is not impaired by DPP1 inhibition or NSP deletion

**FIGURE 5:**
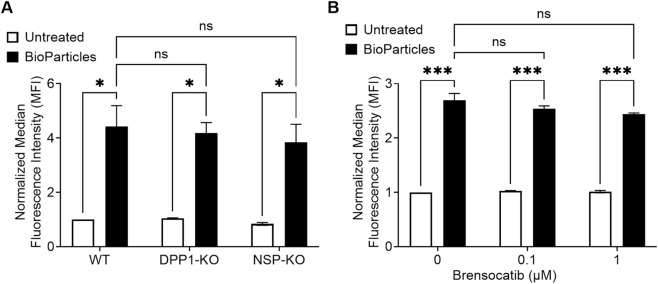
Genetic ablation of DPP1 or of all three major NSPs, or DPP1 inhibition, does not affect mouse neutrophil phagocytosis. **(A)** Neutrophils isolated from the BM of WT, DPP1-KO, or triple NSP-KO mice were incubated (30 min, 37 °C) with pHrodo-labeled bioparticles; uptake of the latter was then analyzed by flow cytometry. **(B)** Mouse BM progenitor cells from WT animals were treated with brensocatib on days 0, 3, and 5 at the indicated concentrations, or with vehicle control, while differentiating into neutrophils. On day 7, neutrophils were incubated with fluorescent bioparticles, and uptake of the latter was analyzed by flow cytometry, as described above. Data is mean ± SEM from 3 independent experiments. Statistical analysis by two-way ANOVA. *p < 0.05, ***p < 0.001.

We then examined the impact of DPP1 or NSP deletion, or of DPP1 inhibition on neutrophil phagocytosis. Mouse BM isolated neutrophils were co-incubated with pHrodo-labeled bioparticles, and their uptake was analyzed by flow cytometry. As shown in Figure 5A, increased intracellular fluorescence was detected 30 min after the addition of the bioparticles, indicating active phagocytosis. No significant differences in fluorescence intensity were observed among neutrophils from the WT, DPP1-KO, and triple NSP-KO groups. Similarly, treatment with brensocatib at concentrations of 0.1 µM or 1 µM for 7 days did not affect the uptake of fluorescent bioparticles by neutrophils differentiated from mouse BM HSCs (Figure 5B). To assess the effect of DPP1 inhibition on human neutrophil phagocytosis, hPMNs were pre-treated with brensocatib for 30 min prior to bioparticle exposure and analyzed by flow cytometry. Consistent with our mouse data, brensocatib treatment did not alter phagocytic activity (Supplementary Figure S4). We finally explored whether DPP1 or its substrates may participate in bacterial killing despite an intact uptake. As shown in Supplementary Figure S5A, the bactericidal activity of mouse neutrophils towards *E.coli* did not significantly differ between WT or DPP1 KO animals. Collectively, these findings indicate that inhibition of DPP1 or of NSP activity does not impair neutrophil phagocytosis or microbicidal capacity.

### Neutrophil ROS production is not impaired by DPP1 inhibition or NSP deletion

**FIGURE 6:**
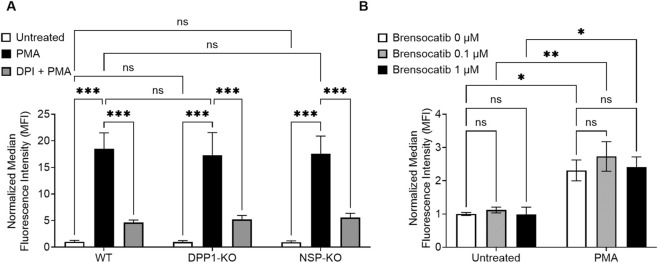
Genetic ablation of DPP1 or of all three major NSPs, or DPP1 inhibition, does not affect mouse neutrophil ROS generation. **(A)** Neutrophils isolated from the BM of WT, DPP1-KO, or triple NSP-KO mice were pretreated with 10 µM DPI or its diluent for 30 min at 37 °C, prior to further incubation with 100 nM PMA or diluent control. ROS production was then assessed by flow cytometry using DHR123. **(B)** Mouse BM progenitor cells from WT animals were treated with brensocatib on days 0, 3, and 5 at the indicated concentrations, or with vehicle control, while differentiating into neutrophils. On day 7, neutrophils were stimulated or not with PMA, and ROS generation was assessed by flow cytometry, as described above. Data is mean ± SEM from 3 independent experiments. Statistical analysis by two-way ANOVA. ***p < 0.001.

Neutrophil ROS production was assessed using dihydrorhodamine 123 (DHR123) under PMA stimulation. Diphenyleneiodonium chloride (DPI), a NADPH oxidase inhibitor (Zavadskis et al., 2020), was used as a control. As shown in Figure 6A, ROS production was dramatically increased in the WT mouse BM isolated neutrophils after 10 min of PMA stimulation; in contrast, DPI pre-treatment significantly suppressed this response. Genetic ablation of either DPP1 or the three major NSPs failed to affect ROS production in mouse neutrophils (Figure 6A). Additionally, treatment with brensocatib during the differentiation of mouse BM-derived neutrophils did not alter ROS generation (Figure 6B). Similar data were obtained in hPMNs, as a 30 min brensocatib pre-treatment had no impact on ROS generation induced by either PMA or N-formylmethionyl-leucyl-phenylalanine (fMLP) (Supplementary Figure S6). These results indicate that inhibiting DPP1 or NSP activity does not impair neutrophil ROS production.

### NET formation is impaired by DPP1 inhibition or NSP deletion in mice

**FIGURE 7:**
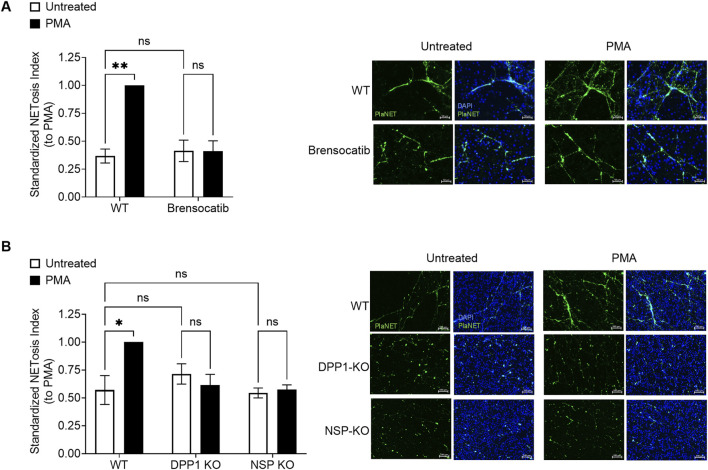
Genetic ablation of DPP1 or of all three major NSPs, or DPP1 inhibition, impairs NET formation in mice. **(A)** Mouse BM progenitor cells from WT animals were treated with 1 µM brensocatib on days 0, 3, and 5 or with vehicle control, while differentiating into neutrophils. **(B)** Likewise, BM progenitor cells from DPP1-KO or triple NSP-KO mice BM progenitor cells were differentiated for 7 days into neutrophils. On day 7, neutrophils from all animals were cultured for 60 min on PLL-coated coverslips and exposed to 100 nM PMA or its diluent (0.1% DMSO) for 4 h. NETosis was then assessed by microscopy using PlaNET Green and DAPI as a nuclear counterstain. Data is mean ± SEM from 3 independent experiments. Statistical analysis by two-way ANOVA. *p < 0.05, **p < 0.01. Representative images of NET formation are shown. Scale bar is 50 µm **(A)** and 100 µm **(B)**.

**FIGURE 8:**
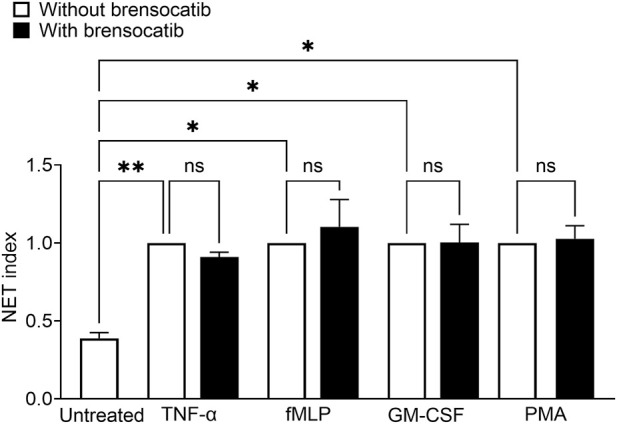
Pharmacological inhibition of DPP1 has no impact on NET formation in hPMNs. hPMNs were isolated from healthy donors, pretreated with brensocatib (1 µM) for 15 min and stimulated with 100 U/mL TNF-α, 100 nM fMLP, 1 nM GM-CSF, or 50 nM PMA for 4 h. NETosis was assessed by microscopy using PlaNET Green and Hoechst 33342 as a nuclear counterstain. Data is mean ± SEM from 4 independent experiments. Statistical analysis by one-way ANOVA. *p < 0.05, **p < 0.01.

Finally, we examined NET generation following DPP1 inhibition or NSP deletion. Mouse BM HSC-differentiated neutrophils were exposed to 100 nM PMA or to a physiological stimulus (fMLP) for 4 h, and NET formation was assessed by microscopy. In WT neutrophils, PMA or fMLP stimulation led to a robust increase in NET formation, as assessed directly by microscopy using PlaNET Green or by quantifying dsDNA from digested NETs (Figure 7, Supplementary Figures S7, S8A). Notably, no such induction of NET generation was observed when DPP1 was inhibited, either via brensocatib treatment during granulocytic differentiation or via genetic ablation. A similar reduction in NET induction was observed in neutrophils from triple NSP-KO mice. These findings suggest that DPP1 and NSP activities are essential during granulocytic differentiation for NET formation to occur in mature mouse neutrophils. To assess the direct impact of DPP1 on NET formation in mature human neutrophils, cells were pretreated with brensocatib prior to stimulation with either TNF-α, fMLP, GM-CSF, or PMA. As shown in Figure 8 and Supplementary Figure S8B, brensocatib had no effect on NET formation in response to any stimulus. We also investigated whether DPP1 or its substrates participate in NET-mediated bacterial killing. As shown in Supplementary Figure S5B, the bactericidal activity of human NETs towards *E.coli* was unaffected by the presence of either brensocatib or combined NSP inhibitors. By contrast, digesting the DNA backbone of NETs with DNase I hindered their ability to kill bacteria (Supplementary Figure S5C), in keeping with the fact that DNA itself can exert antimicrobial actions (Halverson et al., 2015; Ku et al., 2025). The above findings show that DPP1 does not contribute to NET formation or to their antimicrobial action in humans, whether directly or indirectly (through NSP inhibition).

## Discussion

This study provides new insights into the roles of DPP1 and NSPs in neutrophil biology. Through both genetic knockout models and pharmacological inhibition with brensocatib, we confirmed that DPP1 is critical for NSP activation and demonstrated that its inhibition during granulocytic differentiation selectively impairs NET formation in mouse cells, without significantly affecting differentiation itself, or several flagship neutrophil responses.

The finding that DPP1 inhibition with brensocatib, or genetic deletion of DPP1 or NSPs, does not impair neutrophil differentiation aligns with previous observations in patients with Papillon-Lefèvre syndrome (PLS), a rare autosomal recessive disorder characterized by DPP1 deficiency (Pham et al., 2004; Sorensen et al., 2014). PLS patients exhibit normal neutrophil counts despite lacking functional NSPs, suggesting that DPP1 is dispensable for neutrophil development. Similarly, previous studies have shown that DPP1 KO mice exhibit normal immune system function and granulocyte development (Pham and Ley, 1999; Adkison et al., 2002). This suggests that therapeutic targeting of DPP1 does not impair neutrophil production, an essential consideration for its clinical application in NCBF and potentially other inflammatory disorders. Similarly, most of the neutrophil functional responses remained unaffected by DPP1 inhibition or by the deletion of DPP1 or NSPs. First, the neutrophil migratory capacity remained intact, as evidenced both in *ex vivo* transwell chemotaxis assays and in *vivo* inflammatory models. This finding is consistent with previous reports suggesting that NSPs, especially NE, play redundant roles in chemotaxis and *in vivo* recruitment during inflammation (Hirche et al., 2004; Mackarel et al., 1999). This is particularly noteworthy as neutrophil infiltration into sites of infection or inflammation is a critical first step in host defense. The preservation of this function suggests that DPP1 inhibition does not broadly suppress inflammatory responses but rather selectively targets the activation of NSPs. In this regard, our results demonstrate that phagocytosis and ROS production - two key antimicrobial mechanisms deployed by neutrophils–also remain unaffected following DPP1 inhibition or NSP deletion. Accordingly, bacterial killing was similarly unaffected by inhibition or ablation of DPP1 or NSPs. Other possible compensatory mechanisms include the activities of other lytic and bactericidal enzymes. The preserved ability of neutrophils to migrate towards pathogens, as well as to engulf and kill them, highlights the potential of DPP1 inhibition to limit neutrophil-driven tissue damage while maintaining essential host defense mechanisms.

In contrast to all other neutrophil functions examined, NET formation was significantly impaired by DPP1 inhibition or NSP deletion in mice. Whether a similar effect can be observed in humans remains uncertain. In this regard, however, two previous studies from the same group showed that NE participates in NET formation in PMA-stimulated human neutrophils (Metzler et al., 2014; Papayannopoulos et al., 2010). In a subsequent study, we confirmed these findings but also found that unlike PMA, several classes of physiological stimuli could induce NET formation independently of NE in human neutrophils (Tatsiy et al., 2021). Thus, unless NSPs somehow act redundantly towards NET formation (so that blocking only one may not prevent the response), the above considerations collectively indicate a profound difference between mice and humans regarding the participation of NSPs in NET formation. Studies are in progress to further elucidate this possibility.

In summary, our findings demonstrate that while DPP1 and NSPs are essential for NET formation in mice, they are dispensable for other core neutrophil functions, including migration, phagocytosis, ROS production, and bacterial killing, as well as for granulocytic differentiation, in both mice and humans. Our study also reinforces the specificity profile of brensocatib as a therapeutic agent for modulating neutrophil-dependent, inflammation-related tissue damage without compromising antimicrobial defense, as observed in PLS patients (Pham et al., 2004; Sorensen et al., 2014). In keeping with this conclusion, recent clinical trials demonstrated brensocatib’s efficacy in reducing NSP activities and clinical outcomes in patients with NCFB [14, 15]. This raises the possibility that brensocatib treatment may similarly prove beneficial in other neutrophil-driven inflammatory conditions.

## Materials and methods

### Mice

C57BL/6 (WT) mice were purchased from the Jackson laboratory. DPP1-KO and triple NSP-KO mice are under Material Transfer Agreement (MTA) from Washington University, United States. All mice are housed in specific pathogen free conditions under the guidance of the Institutional Animal Care and Use Committee (IACUC) of Rutgers University Animal Care Committee. All animal experiments were conducted under protocols approved by the IACUC of Rutgers University Animal Care Committee.

### Mouse BM HSCs-derived neutrophil differentiation

Mouse BM HSCs were isolated using the mouse BM lineage cell depletion kit following its protocol (Miltenyi, Cat# 130-110-470). Briefly, mouse BM cells isolated from femurs and tibias were incubated with the direct lineage cell depletion cocktail for 10 min. Then the cell suspension was applied to a prepared magnetic separation LS column (Miltenyi, Cat# 130-042-401).

Mouse neutrophil differentiation protocol was slightly modified from the previous study (Gupta et al., 2014). Briefly, BM HSCs were cultured in complete IMDM at 1 × 10^5^ cells/mL with 50 ng/mL SCF (BioLegend # 579706) and 50 ng/mL IL-3 (BioLegend # 575506) for 3 days. Treatment groups received either vehicle or brensocatib at 0.1 µM or 1 µM. Cells were resuspended at 2 × 10^5^ cells/mL in fresh medium supplemented with mouse-SCF, mouse-IL-3, and 50 ng/mL G-CSF (BioLegend, Cat# 574606) on day 3, and cultured for 2 days with fresh treatments. On day 5, cells were washed and resuspended at 5 × 10^5^ cells/mL in G-CSF (50 ng/mL) with treatments and cultured for another 2 days. On day 7, cells were harvested and analyzed by flow cytometry. Neutrophils were identified as CD45^+^CD11b^+^Ly6G^+^, and the gating was based on a quadrant separation relative to the undifferentiated population.

### Isolation of mouse BM neutrophils

Mouse BM neutrophils were isolated using the mouse BM neutrophil isolation kit according to the manufacturer’s protocol (Miltenyi, Cat# 130-097-658). Briefly, mouse BM cells isolated from femurs and tibias were incubated with the neutrophil biotin-antibody cocktail for 10 min. Then cells were incubated with the anti-biotin microbeads for another 15 min. Cell suspension was then applied to prepare magnetic separation LS column (Miltenyi, Cat# 130-042-401). Resuspend the cell pellet with 1 mL ice-cold complete RPMI 1640 medium and the cells are ready for downstream study.

### Isolation of human peripheral blood neutrophils (hPMNs)

Whole blood from healthy donors was collected in citrate phosphate and processed via dextran sedimentation, Ficoll gradient separation, and hypotonic water lysis, as previously described (Ear et al., 2005). All procedures were performed at room temperature under endotoxin-free conditions. Purified neutrophils were resuspended in RPMI 1640 medium with 5% autologous serum at 5 × 10^6^ cells/mL, unless otherwise indicated. Final cell preparations contain >99% hPMNs, confirmed by flow cytometry.

### Human CD34^+^ HSC-derived neutrophil differentiation

Human umbilical cord or BM-derived CD34^+^ stem cells purchased from AllCells were resuspended in STEMSPAN™ SFEM II medium (Stemcell Technologies, Cat# 09655) supplemented with 1× StemSpan™ CD34^+^ Expansion Supplement (Stemcell Technologies, Cat# 02691), and cultured at 37 °C for 7 days. After 7 days of expansion, cells were pelleted and resuspended in fresh SFEM II medium supplemented with 100 ng/mL rh-SCF (BioLegend, Cat# 573906), 100 ng/mL rh-IL-3 (BioLegend, Cat# 578006), and 10 ng/mL rh-G-CSF (BioLegend, Cat# 578606). Treatment groups received vehicle or brensocatib at 0.1 µM, or 1 µM. Cells were cultured for an additional 9 days, with medium and treatments renewal every 2–3 days. On day 9, neutrophil maturation was assessed by flow cytometry.

### DPP1 and NSP enzymatic activities measurement

Mouse or human cell lysates were prepared and DPP1 and NSP activities were measured as previously described (Cipolla et al., 2023; Chen et al., 2023; Sutton et al., 2024; Basso et al., 2022). Briefly, substrates for DPP1 (H-Gly-Arg-AMC; Bachem # 4002196.0050), NE (MeOSuc-AAPV-AMC; Sigma #M9771), PR3 (7-Methoxycoumarin-4-yl)acetyl-lysyl-(picolinoyl)-Tyr-Asp-Ala-Lys-Gly-Asp-N-3-(2-4-dinitrophenyl)-2-3-diaminopropyonyl-NH2 for mouse; Abz-VADCADQ-Lys(DNP) for human; GenScript custom synthesis), and CatG (Suc-AAPF-pNA; Sigma S7388) were co-incubated with the diluted cell lysates. Fluorescence or absorbance was recorded using a synergy microplate reader. Specific DPP1 or NSP activity was calculated by subtracting activity in the presence of inhibitors from total activity. Concentrations were interpolated from standard curves using active DPP1, NE, PR3, and CatG proteins. Total protein was measured with a Pierce™ BCA Protein Assay Kit (Thermo Fisher, Cat# A654453). DPP1 and NSP activities were normalized accordingly.

### Transwell migration assay

Mouse or human neutrophils were loaded in the upper chamber of 3-µm pore size transwell plates, with 100 ng/mL CXCL2 (mouse; BioLegend, Cat# 582504) or CXCL8 (human; BioLegend, Cat# 574204) in the lower chamber and 2 μg/cm^2^ laminin on the insert. Cells were incubated at 37 °C for 30 or 60 min, and neutrophil migration rates were calculated as the cell count in the lower chamber/load control × 100%.

### Mouse acute lung injury model

To evaluate inflammatory responses in WT and DPP1-KO mice, an intratracheal (IT) LPS challenge was employed. LPS (*Escherichia coli* O111:B4, Sigma, Cat# L3024) was prepared in PBS fresh on the day of dosing and administered IT at 3 mg/kg in a 50 µL volume. Mice were anesthetized with 2.5% isoflurane in oxygen and placed in supine position on a sloped intubating platform. A micro sprayer was inserted into the trachea to deliver aerosolized LPS. Mice were returned to cages for recovery post dosing. At 24 h post-LPS challenge, bronchoalveolar lavage (BAL) samples were collected in two sequential PBS lavages (800 µL followed by 600 µL), with immediate withdrawal after each instillation. Recovered fluids were pooled and kept on ice until same-day processing.

### The MSU crystal-induced air pouch model

The MSU crystal-induced air pouch model was developed as previously described (Devi et al., 2024). MSU crystals (InvivoGen, Cat# tlrl-msu) at 5 mg/mL in PBS were used to induce inflammation in the dorsal air pouch model. On day 0, mice were weighed, shaved, and injected subcutaneously with 5 mL sterile air over the dorsum to form an air pouch. Pouches were reinflated on day 3 with 3 mL sterile air. On day 6, pre-warmed MSU suspension (1 mL per mouse) or PBS (control) was injected into the pouch. Mice were then euthanized by CO_2_ asphyxiation after 5 h, and pouch lavage was performed with 2 mL PBS. Lavage fluid was gently mixed and kept on ice for further analysis.

### Cytokine measurement

Cytokine levels were measured using the Bio-Plex Pro™ Mouse Cytokine Th1 Panel (Bio-Rad, Cat#L6000004C6) according to the manufacturer’s protocol. Briefly, coupled magnetic beads were diluted 1:10 in assay buffer (included in the kit), vortexed, and 50 µL added per well, followed by two washes with wash buffer (included in the kit). Then, 50 µL of standards or samples were added to each well. Plates were sealed, incubated for 30 min at room temperature (RT) with shaking (850 rpm), protected from light, and washed three times. Twenty-5 µL of 1X detection antibody was added per well, followed by a 30-min RT incubation with shaking. After washing, 1X streptavidin-PE (SA-PE) was prepared from 100X stock, and 50 µL was added per well, incubated for 10 min at RT with shaking. Plates were washed again and resuspended in 125 µL of assay buffer, then read using the Bio-Plex 200 System (low PMT, RP1 settings). Cytokine concentrations were calculated using Bio-Plex Manager Software v6.1 (Bio-Rad) with a five-parameter logistic (5 PL) regression model.

### Neutrophil phagocytosis assay

Mouse or human neutrophils were diluted to 1 × 10^6^ cells/mL and 50 µL was added per well in a 96-well flat-bottom plate. Cells were incubated with the vehicle or brensocatib at 0.1 µM, or 1 μM at 37 °C for 30 min pHrodo™ Green *E. coli* BioParticles™ (ThermoFisher #P35366) were reconstituted in 2 mL Opti-Klear™ Live Cell Imaging Buffer (Abcam t# ab275938) to a concentration of 1 mg/mL, vortexed, and sonicated for 10 min to disperse particles. For the assay, this suspension was diluted 1:5 in the imaging buffer to obtain a 200 μg/mL working stock. Fifty µL of this suspension was added. Plates were incubated at 37 °C (humidified incubator, with shaking) for 30 or 60 min to allow phagocytosis, while control plates were incubated at 4 °C. To assess phagocytosis and viability, 1 µL of propidium iodide (PI) was added per well, and samples were analyzed by flow cytometry using the FITC channel for pHrodo Green fluorescence and the PE channel for PI viability staining.

### ROS measurement

Mouse or human neutrophils were diluted to 0.5 × 10^6^ cells/mL, and 100 µL of cells were seeded into a 96-well flat-bottom plate. The cells were incubated with either vehicle or brensocatib at 0.1 µM or 1 µM. For negative controls, DPI (10 μM; Sigma #D2926) was added, followed by a 30-min incubation at 37 °C. The cells were then stimulated with PMA (100 nM; Sigma, Cat# P1585) or fMLP (100 nM; Sigma #F3506). DHR123 (Sigma #D1054) was added at a final concentration of 20 µM together with the stimuli. The cells were incubated for 10 min at 37 °C on a shaker (100 rpm), then washed and resuspended in 150 µL PBS. Propidium iodide (2 µL) was added per well, and samples were analyzed by flow cytometry using the FITC channel for DHR123 and the PE channel for PI viability staining.

### NETosis assay

The NET microscopic assay was performed as previously described with slight modifications (de Carvalho Oliveira et al., 2023). In brief, 500 µL of mouse neutrophils (0.5 × 10^6^ cells) or hPMNs (1 × 10^6^ cells) were seeded onto poly-L-lysine (PLL)-coated coverslips in a 24-well plate and allowed to adhere for 60 min at 37 °C. Cells were pre-treated (15 min, 37 °C) with vehicle or brensocatib at 0.1 µM or 1 μM, followed by stimuli for 4 h. Reactions were stopped by incubating cells with 500 µL ice-cold PBS containing 1 mM of phenylmethanesulfonyl fluoride (PMSF, 100 mM; Sigma #P7626) on ice for 10 min. The reagent was then discarded and replaced by 500 µL ice-cold PBS containing 1 mM of PMSF and diluted PlaNET reagent (PolySciences #16661-10) for NET detection. Cells were incubated with PlaNET reagent for 90 min on ice with gentle shaking. Then, the cells were washed with PBS and fixed with 4% paraformaldehyde containing DAPI (1:500) for 15 min. The fixed cells were washed once with PBS and mounted with ProLong Gold. Images were acquired the next day using a Zeiss Apotome fluorescence microscope (×10 objective); three representative images per well were analyzed using a PlaNET ImageJ plugin (available at: http://mcdonaldlab.ca/java-plug-in.html), excluding edge-adhered cells, clumps, or abnormal staining.

### Generation of human NET fragments

Neutrophils were made to adhere on PLL-coated coverslips as described above for NETosis assays, prior to a 4-h stimulation with 1 μg/mL LPS (Sigma #L4391). Thereafter, some coverslips were set aside to ascertain microscopically that NET formation had occurred in that experiment (as described for NETosis assays). For all other wells, culture supernatants were gently aspirated and discarded and the remaining layer, containing NETs and intact neutrophils, was covered with 250 µL/well of NET collection buffer (10 mM TrisBase pH 8.0, 2.5 mM MgCl_2_, 0.5 mM CaCl_2_ and 10 U/mL MNase (ThermoFisher #EN0181)) prior to a 30-min incubation at 37 °C to digest the immobilized NETs. Reactions were terminated by placing cells on ice for 15 min. NET supernatants were then gently collected, transferred into 50-mL polypropylene tubes, and centrifuged (400 g, 5 min, 4 °C) to remove any cell debris. The resulting supernatants, containing mostly NET fragments, were aliquoted in DNA LoBind tubes (Eppendorf #13-698-791), snap-frozen in liquid nitrogen, and stored at −80 °C until further use.

### Quantification of dsDNA in digested NETs

The concentration of dsDNA in isolated NET fragments (prepared as described above) was determined using the Quant-iT PicoGreen dsDNA Assay Kit (ThermoFisher #P11496) according to the manufacturer’s instructions. Fluorescence was measured in a Qubit Fluorometer (ThermoFisher #Q32857) or BioTek Synergy Neo reader (Agilent Technologies).

### Bacterial killing assays

*Escherichia coli* (DH5α; NEB #C2987H) were grown to exponential phase and quantified using an OD_600_-CFU standard curve. For neutrophil killing assays, BM-derived neutrophils from WT or DPP1-KO mice were plated at 1 × 10^5^ cells per well in HBSS containing Ca^2+^/Mg^2+^, primed with TNF-α (20 ng/mL; R&D Systems #410-MT) and GM-CSF (50 ng/mL; Biolegend #576306) for 45 min and then infected with *E. coli* opsonized with mouse serum for 15 min at 37 °C at an MOI (Multiplicity of infection) of 0.2 for 1 h at 37 °C. Reactions were terminated by saponin lysis (∼0.1%), and bacterial survival was quantified by CFU enumeration.

For NET-mediated killing assays, human NETs were incubated with *E. coli* at an MOI of 0.025 (normalized to neutrophil input; 250 µL NETs collected per 1 × 10^5^ human neutrophils) for 1 h at 37 °C. Where indicated, hNETs were pretreated for 10 min with either a combination of 10 µM cathepsin G inhibitor (Cayman #14928) and 10 µM sivelestat (Sigma S7198), or with 1 µM brensocatib. Wells containing NET buffer only, or bacteria only, served as controls. Bacterial killing was calculated relative to the bacteria-only control condition.

### LDH activity measurement

LDH activity in lavage fluid supernatants was quantified using the LDH-Glo Cytotoxicity Assay (Promega #J2381) according to the manufacturer’s instructions. Clarified supernatants were added to white 96-well plates, followed by an equal volume of freshly prepared LDH Detection Reagent. After incubation for 60 min at room temperature protected from light, luminescence was measured in a microplate reader.

### Flow cytometry

Staining of mouse neutrophils was performed with surface antibodies including CD45 (BioLegend #103108), CD11b (BioLegend #101222), and Ly6G (BioLegend t#127608). Staining of hPMNs was performed with surface antibodies including CD11b, CD66b (BioLegend #305106), and CD16 (BioLegend #302006). Dead cells were excluded using the Live/Dead dye (Invitrogen #L34964). The samples were acquired on an Attune NxT flow cytometer and analyzed with FlowJo software.

### Statistical analyses

All data are expressed as mean ± SEM. Any outlying data point (as determined by Grubb’s test) was excluded when computing means. Statistical analyses were performed using the analysis of variance (ANOVA), log-rank, Mann-Whitney, or Student’s t-test using GraphPad Prism 10 software. *p < 0.05; **p < 0.01; ***p < 0.001.

## Data Availability

The raw data supporting the conclusions of this article will be made available by the authors, without undue reservation.
