# Supplementary material for: Impact of dipeptidyl peptidase I and neutrophil serine proteases on neutrophil functional responses
Source: Front Pharmacol. 2026 Apr 24;17:1689804. doi: 10.3389/fphar.2026.1689804 (PMC13152848; doi:10.3389/fphar.2026.1689804)
Supplement: Supplementary file 1 [file Supplementaryfile1.docx]

Supplementary Material

## Supplementary Figures


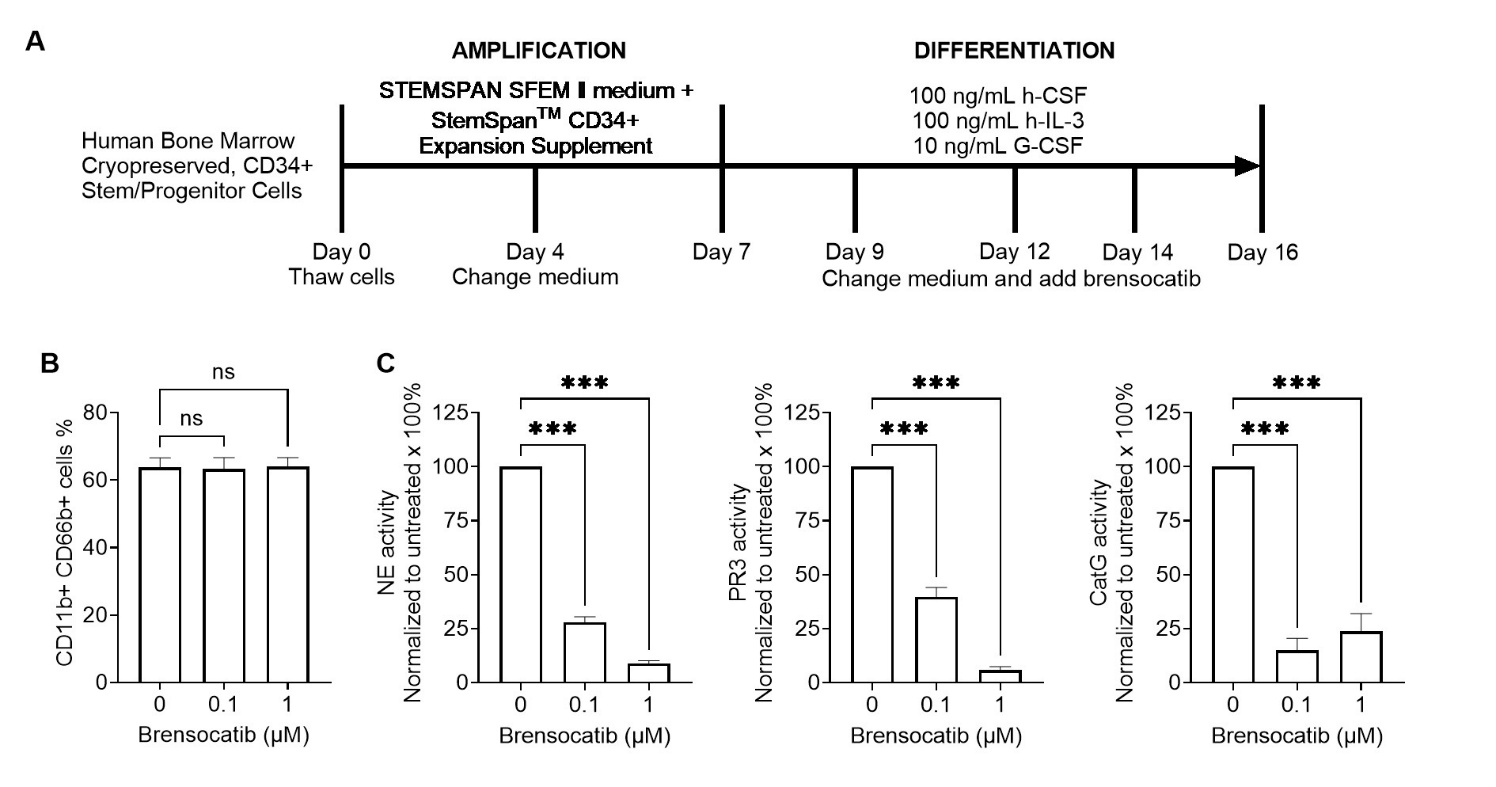


**Supplementary Figure 1.** **Human neutrophil differentiation is not affected by a 9-day treatment with brensocatib. (A)** Protocol for neutrophil differentiation from human CD34^+^ stem cells (HSCs). HSCs were differentiated into neutrophils in the presence of 0.1 or 1.0 µM brensocatib (or its diluent) starting on day 7; culture medium was refreshed every 2-3 days. Cells were collected on day 16, neutrophil surface markers were analyzed **(B)**, and NSP enzymatic activities in the cell lysates were measured **(C)**. Statistical analysis was conducted using one-way ANOVA (B).

**
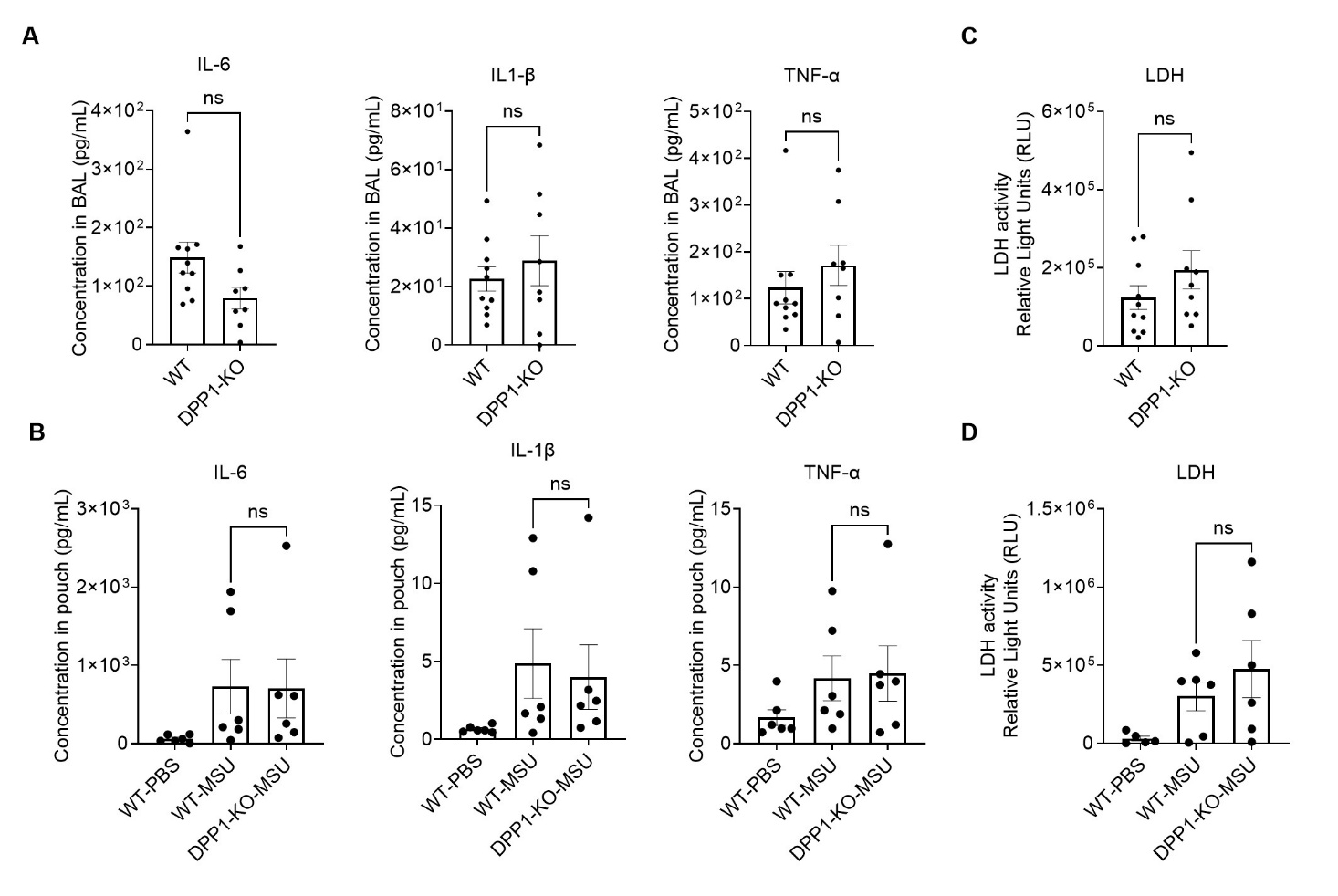
**

**Supplementary Figure 2.** **Pro-inflammatory cytokine (IL-6, IL-1β, and TNF-α) expression and LDH activity at the site of inflammation in the LPS intratracheal challenge mouse model (A, C) and the MSU-induced dorsal air pouch mouse model (B, D).** Statistical analyses were performed using Student’s t test (A, C) or two-way ANOVA (B, D).

**
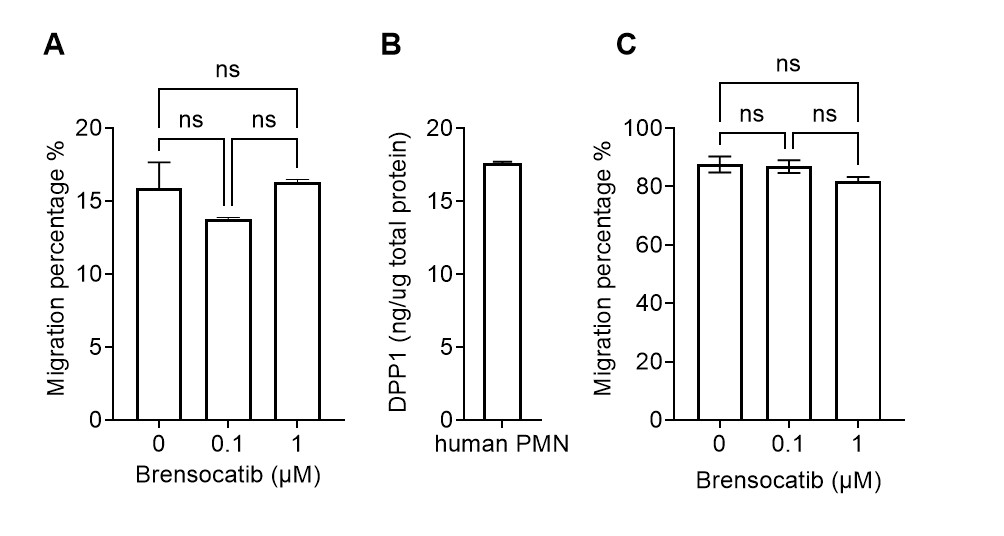
**

**Supplementary Figure 3. Brensocatib does not affect human neutrophil migration. (A)** Human CD34^+^ HSCs were differentiated into neutrophils. Different doses of brensocatib were added on day 7 and refreshed every 2-3 days. Cells were collected on day 16, and neutrophil migration was evaluated using the transwell migration assay. (**B**) Human peripheral blood neutrophils were isolated and DPP1 enzymatic activity in the cell lysates was measured. (**C**) Human peripheral blood neutrophils were pre-treated with brensocatib for 30 minutes. Neutrophil migration was evaluated using the transwell migration assay. Statistical analysis was conducted using one-way ANOVA.


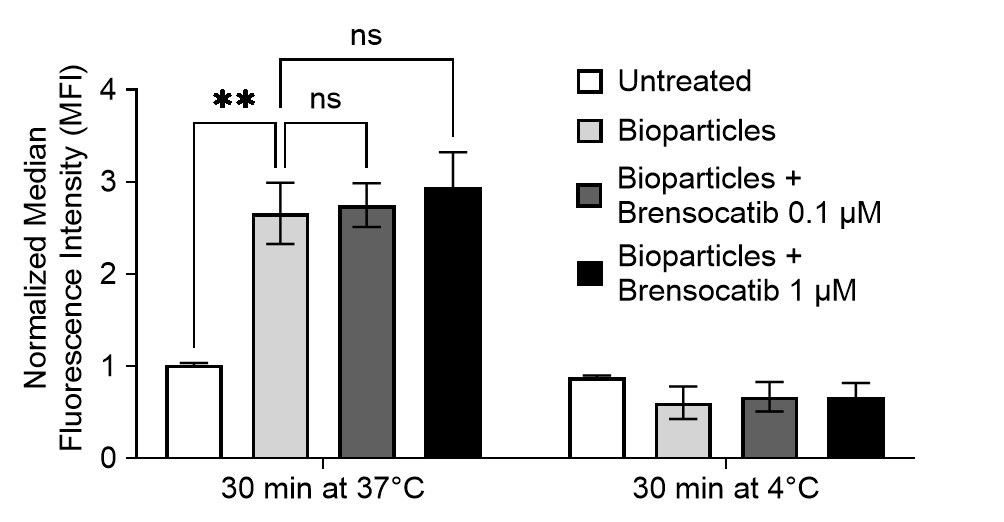


**Supplementary Figure 4. Brensocatib does not affect human neutrophil phagocytosis.** Human peripheral blood neutrophils were pre-treated with brensocatib for 30 minutes. The cells were then incubated (37°C or 4°C) with pHrodo-labeled bioparticles for 30 minutes. Neutrophil phagocytosis was then analyzed by flow cytometry. Statistical analysis was conducted using two-way ANOVA. Mean ± SEM from 3 independent experiments. **p < 0.01.


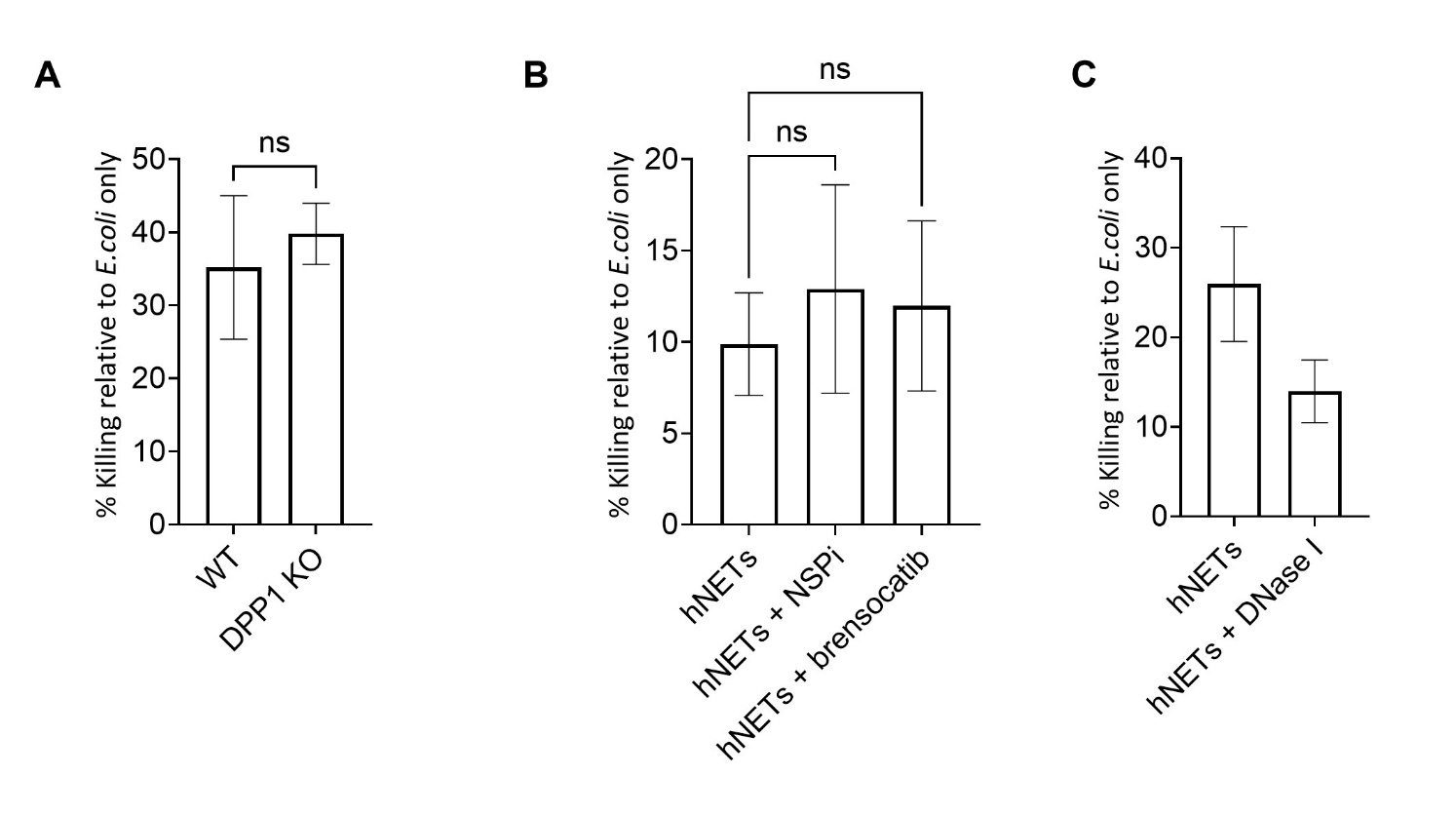


**Supplementary Figure 5. Genetic ablation of DPP1, or pharmacological inhibition of DPP1 or of the three major NSPs, does not impair the bactericidal activity of mouse neutrophils or of human NETs.** (**A**) BM neutrophils from WT and DPP1 KO mice were isolated and co-incubated with opsonized *E. coli* for 60 minutes. Bactericidal activity was calculated as the percentage of CFUs recovered from wells containing neutrophils relative to the CFUs from *E. coli*–only control wells. Mean ± SEM from 3 independent experiments. (**B**) Human NETs were co-incubated with *E. coli* for 60 min in the presence of 1 µM brensocatib, a combination of 10 µM sivelestat + 10 µM CatG inhibitor (“NSPi”), or their diluent (0.2% DMSO, final concentration). Bactericidal activity was then calculated as the percentage of CFUs recovered from NET-containing wells, relative to *E. coli*–only controls. Mean ± SEM from 3 independent experiments. (**C**) Human NETs were pre-treated or not with 100 U/ml DNase I for 30 min and then co-incubated with *E. coli* for 60 min. Bactericidal activity was then calculated as in (B). Mean ± SEM from 2 independent experiments.





**Supplementary Figure 6. Pre-treatment with brensocatib does not affect PMA/fMLP-induced ROS production in hPMNs. (A)** PMA or fMLP significantly induced ROS production in hPMNs. hPMNs were isolated from healthy donors and stimulated with PMA or fMLP for 10 minutes. ROS production was measured using DHR123. **(B)** Brensocatib did not affect hPMNs ROS production. hPMNs were pre-treated with/without brensocatib at the indicated concentrations for 30 minutes. Cells were then stimulated with PMA for 10 minutes. ROS production was measured using DHR123. Statistical analyses were conducted using one-way ANOVA (A) or two-way ANOVA (B). *p < 0.05, **p < 0.01, ***p < 0.001.


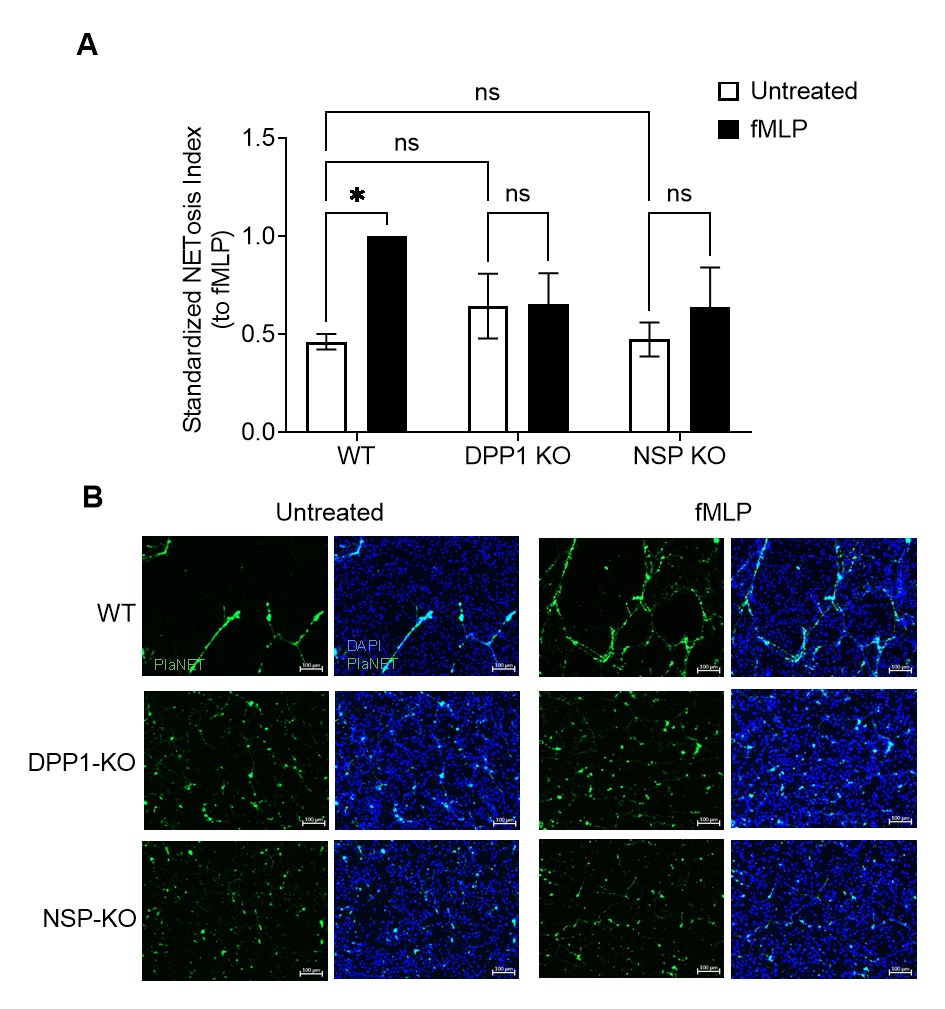


**Supplementary Figure 7. Genetic ablation of DPP1 or NSPs impairs NET formation in mice.** **(A)** BM progenitor cells from WT, DPP1 KO or triple NSP KO mice were differentiated for 7 days into neutrophils. On d7, neutrophils from all animals were cultured for 60 minutes on PLL-coated coverslips and exposed to 100 nM fMLP or its diluent (0.1% DMSO) for 4h. NETosis was assessed by microscopy using PlaNET Green; DAPI was used to stain cell nuclei. Statistical analysis was conducted using two-way ANOVA (A). Mean ± SEM from 3 independent experiments. *p < 0.05. **(B)** Representative images of NET formation. Scale bar is 100 µm.


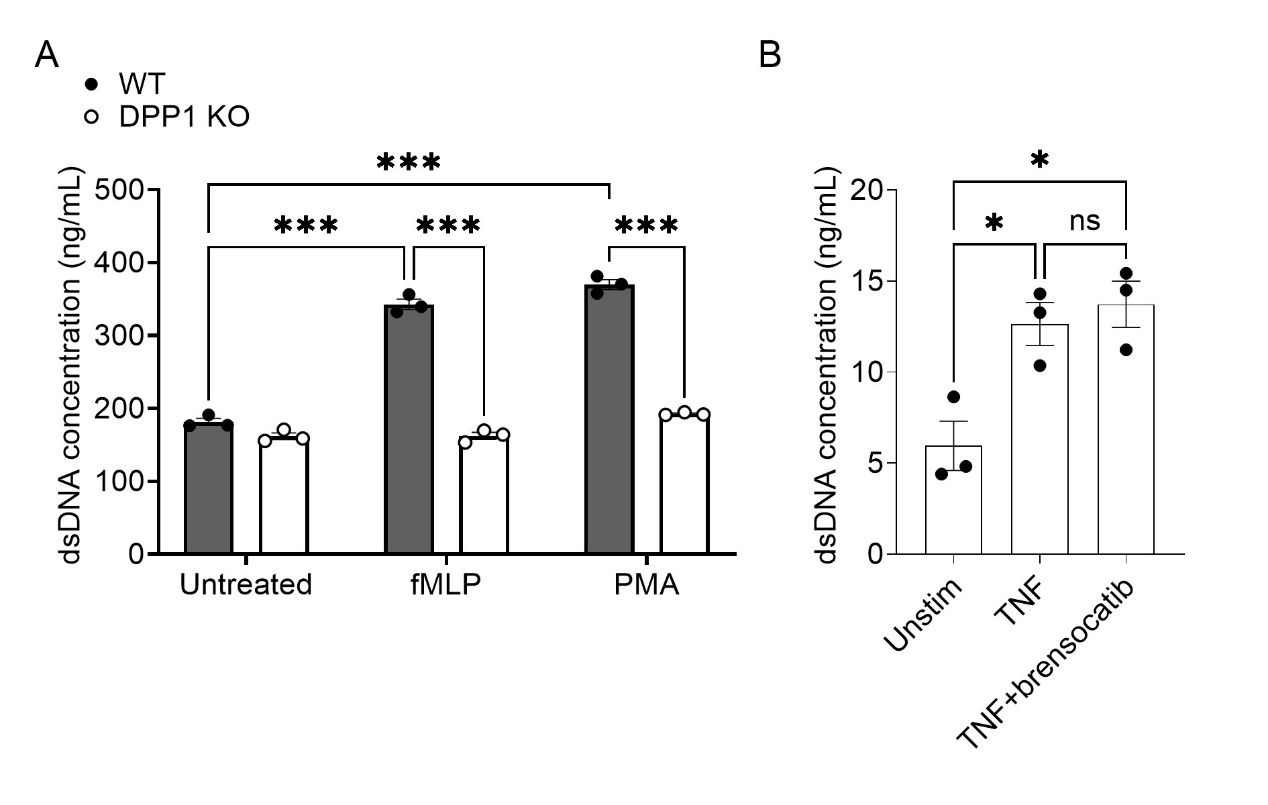
**Supplementary Figure 8. NET quantification as determined by dsDNA measured in digested NETs. (A)** BM progenitor cells from WT or DPP1 KO mice were differentiated for 7 days into neutrophils. On d7, neutrophils were cultured for 60 min on PLL-coated coverslips and exposed to 100 nM PMA or fMLP, or their diluent (0.1% DMSO), for 4 h. Culture medium was removed and adhered material (i.e. intact cells and NETs) was submitted to MNase digestion. The resulting supernatants (containing NET fragments) were then assessed for dsDNA content. **(B)** hPMNs were isolated from healthy donors, pretreated with brensocatib (10 µM) for 15 min, and stimulated with 100 U/mL TNF-α for 4 h. NET quantitation was then performed as described in (A). Statistical analyses were conducted using two-way ANOVA (A) or one-way ANOVA (B). Mean ± SEM from 3 independent experiments. *p < 0.05, ***p < 0.001.
